# Comparing genomes recovered from time-series metagenomes using long- and short-read sequencing technologies

**DOI:** 10.1186/s40168-023-01557-3

**Published:** 2023-05-13

**Authors:** Luis H. Orellana, Karen Krüger, Chandni Sidhu, Rudolf Amann

**Affiliations:** Department of Molecular Ecology, Max Planck Institute for Marine Microbiology, Celsiusstraße 1, Bremen, 28359 Germany

**Keywords:** Long read sequencing, Metagenome-assembled genomes, Metagenomic sequencing

## Abstract

**Background:**

Over the past years, sequencing technologies have expanded our ability to examine novel microbial metabolisms and diversity previously obscured by isolation approaches. Long-read sequencing promises to revolutionize the metagenomic field and recover less fragmented genomes from environmental samples. Nonetheless, how to best benefit from long-read sequencing and whether long-read sequencing can provide recovered genomes of similar characteristics as short-read approaches remains unclear.

**Results:**

We recovered metagenome-assembled genomes (MAGs) from the free-living fraction at four-time points during a spring bloom in the North Sea. The taxonomic composition of all MAGs recovered was comparable between technologies. However, differences consisted of higher sequencing depth for contigs and higher genome population diversity in short-read compared to long-read metagenomes. When pairing population genomes recovered from both sequencing approaches that shared ≥ 99% average nucleotide identity, long-read MAGs were composed of fewer contigs, a higher N50, and a higher number of predicted genes when compared to short-read MAGs. Moreover, 88% of the total long-read MAGs carried a 16S rRNA gene compared to only 23% of MAGs recovered from short-read metagenomes. Relative abundances for population genomes recovered using both technologies were similar, although disagreements were observed for high and low GC content MAGs.

**Conclusions:**

Our results highlight that short-read technologies recovered more MAGs and a higher number of species than long-read due to an overall higher sequencing depth. Long-read samples produced higher quality MAGs and similar species composition compared to short-read sequencing. Differences in the GC content recovered by each sequencing technology resulted in divergences in the diversity recovered and relative abundance of MAGs within the GC content boundaries.

**Supplementary Information:**

The online version contains supplementary material available at 10.1186/s40168-023-01557-3.

## Background

In shotgun metagenomic approaches, limitations in the read length (i.e., ~ 100–250 bp sequences) often translate into fragmented reconstructed metagenome-assembled genomes (MAGs) with uncertain levels of genomic completion during de novo assembly. These complications are primarily due to highly repetitive regions, high levels of sequence microdiversity, multiple copies of genes, and AT-rich/GC-rich regions [1]. Overcoming these limitations is paramount to understanding the role of microorganisms in natural processes and analyzing their diversity in environmental and gut microbiomes.

The emergence of long-read sequencing technologies restores the hopes of overcoming these limitations in genomic sequence recovery. Sequencing platforms from Oxford Nanopore and Pacific Biosciences (PacBio) can produce longer reads, although at the expense of a higher sequencing error rate and less sequencing depth compared to Illumina short-reads [2] (SR). For instance, the median length of reads ranges from 5 to 20 kbp and throughputs from 15 to 50 Gbp in LR technologies. Current sequencing chemistries yield observed modal read accuracies of 99.99%, 99.14%, and 99.9% for Illumina, Oxford Nanopore, and PacBio, respectively [3, 4]. Moreover, advances in technological and bioinformatic approaches are closing the gaps between short- and long-read sequencing technology applications, especially for recovering high-quality MAGs from the environment. Thus, long-read (LR) shotgun metagenomics is poised to set new standards for MAG quality. For instance, current PacBio Sequel II technology offers circular consensus sequencing (CCS), providing a low-error rate in high fidelity reads, although at a shorter read length than the traditional long-read technology [3]. Additionally, better genome statistics (low number of contigs and high N50 values) [5–9] or the combination of short- and long-reads for the recovery of high-quality MAGs [10, 11] have already showcased the benefits for the recovery of microbial genomes [12]. Nonetheless, whether a switch from SR to LR metagenomic approaches would introduce biases for capturing the genetic potential of microbial populations in terms of recovery of the MAGs is less known.

## Methods

### Sampling and sequencing

During the spring of 2020, surface seawater samples were collected from the “Kabeltonne” long-term ecological research station off the North Sea of Helgoland (54° 11.3′ N, 7° 54.0′ E) as described previously [13, 14]. Surface water samples were collected on March 10, 2020, April 14, 2020, April 30, 2020, and May 6, 2020. All samples were filtered through 10- and 3-μm pore-size filters to remove phytoplankton and particle-associated microorganisms. Cells were collected using 0.2-μm pore-size polycarbonate filters, and DNA extractions were performed using the ZR-Duet DNA/RNA MiniPrep Plus kit (Zymo Research). DNA extractions were split for Illumina and PacBio, and all eight samples were individually sequenced (not multiplexed) at the Max Planck Genome Centre in Cologne, Germany. Sequencing was performed on an Illumina HiSeq 2500 (rapid mode) and a PacBio Sequel II platform. High-fidelity reads (HiFi) were generated using the circular consensus method (Table S1). Trimming and processing of raw Illumina reads were performed using BBduk v38.94 (BBtools; http://bbtools.jgi.doe.gov; settings: ktrim = r, k = 28, mink = 12, hdist = 1, tbo = t, tpe = t, qtrim = rl, trimq = 20, minlength = 100). Read quality assessments were performed using FASTQC v0.11.9 (www.bioinformatics.babraham.ac.uk/projects/fastqc/) and Nanoplot [15] v1.32.1. The average sequence coverage was determined using Nonpareil [16] v3.304. Since Nonpareil was not originally designed to be used with LRs, we used single ~ 250 bp fragments generated from each read contained in LR metagenomes (i.e., unique fragments per long read). LRs were cut into shorter sequence fragments using the shred.sh script from BBtools (length = 250 minlength = 100), and one random fragment was selected from each LR for further analyses. Thus, for LR metagenomes, the average sequence coverage was predicted using the Nonpareil model and the original sequencing effort (lr) for each sample in the “predict.Nonpareil.curve” algorithm.

### Assembling, binning, abundances, and taxonomy of metagenome-assembled genomes

The assembly and binning of metagenome-assembled genomes (MAGs) using Illumina metagenomic samples were carried out as described previously [17]. First, contig sequence statistics were determined using the scripts Fasta.N50.pl, FastA.gc.pl, and FastA.qlen.pl from the enveomics collection [18]. The assemblies of SR were generated using SPAdes [19] v3.14.1 (meta option, kmers = 21,33,55,77,99, and 127), and contigs longer than 2.5 kbp were binned using CONCOCT [20] v1.1.0, MaxBin2 [21] v2.2.7, Metabat2 [22] v2.12.1, Binsanity [23] v0.4.4, and integrated using DAS_Tool [24] v1.1.2. Long-read metagenomic samples were assembled using Flye [25] (meta option) v2.8, and resulting contigs longer than 2.5 kbp were binned using Metabat2 v2.12.1 within anvi’o v6.2 [26]. Hybrid assemblies were performed using hybridSPAdes [27] as implemented in SPAdes v3.15.5 using the same configuration above but providing the corresponding LR library (–pacbio). Bins from SR and LR technologies were also quality-refined in anvi’o v6.2 [26]. Sequences encoding 16S rRNA genes were detected using barrnap v0.9 (https://github.com/tseemann/barrnap). The mapping of LR for calculating sequencing depth needed for the binning of contigs and MAG abundance was performed using pbmm2 (https://github.com/PacificBiosciences/pbmm2/; --preset HIFI -× 97 -N 1), which is a wrapper for minimap2 [28]. All MAGs were filtered based on a quality metric based on completion and contamination values obtained from checkM [29] v1.1.3 ([completion%] - 5*[contamination%] >  = 50). The de-replication of MAGs was done to assess the number of MAGs sharing > 99% ANI obtained from each sequencing platform using dRep [30] v3.0.0. For statistical tests between pairs of MAGs, the Shapiro-Wilk normality test and Wilcoxon rank tests were performed in the R statistical software v4.1.1.

For Illumina metagenomes, MAG abundances were determined as relative abundance (mapped reads/total reads) and as the quotient between the truncated average sequencing depth (TAD) [31] and the total sequencing depth of microbial genomes “genome equivalents” as determined in MicrobeCensus [32] v1.1.1. The truncated average sequencing depth was determined using BedGraph files considering zero-coverage positions (bedtools genomecov -bga) [33] and the “BedGraph.tad.rb” script (-r 0.8) from the enveomics collection [18]. Abundances for MAGs derived from LR were determined using the average sequencing depth (i.e., non-truncated) as specified above and normalized using the median sequencing depth of 16 single-copy gene markers predicted in unassembled long-reads (rpl2, rpl3, rpl4, rpl5, rpl6, rpl14, rpl15, rpl16, rpl18, rpl22, rpl24, rps3, rps8, rps10, rps17, and rps19; see gene prediction and annotation below).

MAGs defined as “shared” or detectable using both technologies were defined as those MAGs sharing >  = 99% ANI, as determined in fastANI [34] v1.32, obtained from each technology at one specific sampling date. Taxonomic classification of MAGs was performed using GTDB-tk [35] v1.7 and the GTDB [36] release r202. In GTDB-tk, MAGs are classified into species using a 95% ANI threshold.

### Comparison of gene predictions in unassembled and assembled long-read metagenomes

Gene predictions in unassembled LR were performed using FragGeneScan [37] v1.31. However, we compared different tools to ensure better gene predictions. First, for the March 10, 2020, LR sample gene predictions were performed using Prodigal [38] v2.6.3 (meta option), MetaGeneMark [39] v3.38, and FragGeneScan [37] v1.31. For the last algorithm, we compared predictions using complete/short sequences (-w 0 or 1) and different sequencing error models (sanger_5 and sanger_10). All predicted sequences were compared against the TrEMBL protein sequence database (downloaded April 27, 2021) using DIAMOND [40] BLASTp v2.0.8.146 (--max-target-seqs 5). Best matches were selected based on the highest bitscore value, and the query lengths longer than 100 amino acids, *e* value ≥ 1e^−10^, and sequence identity ≥ 40% were compared against its match reference length.

### Code availability

Code, pipelines, and analyses are available on GitLab at https://gitlab.mpi-bremen.de/lorellan/ilmn-vs-pacb-helgoland.

### Data availability

Metagenomes and MAGs data are available from the European Nucleotide Archive (ENA) project PRJEB52999 [14].

## Results and discussion

### Unassembled read statistics

We sequenced DNA extracted from bacterioplankton collected in the 0.2–3 μm size fractions at four time points during the spring of 2020 in the North Sea. The sampling points comprised one pre- and three bloom events, part of a time-series sampling campaign during 2020 [14]. The Illumina SR approach recovered an average of 36 Gbp per sample (~ 240 bp paired-end reads), while the CCS PacBio LR approach recovered an average of 12 Gbp per sample (~ 6 kbp CCS reads) (Table S1). This difference translated into SR metagenomic samples having, on average, ~3 times more sequenced base pairs than LR samples, which is within the expected output for single sequenced samples [8]. Additionally, LR metagenomic samples had slightly higher GC content (0.43) compared to their SR counterpart (0.38) when all reads were considered (Table S1). The average coverage of the microbial community was overall similar between LR metagenomes (not significantly different; Fig. S1) as determined in Nonpareil [16]. The main difference was the LR sample from April 30, 2020, had a comparatively lower sequencing depth than other LR samples. Similarly, when determining sequence diversity using a combined measure of richness and evenness (total diversity), LR metagenomes had higher average sequence diversity, although not significantly different from SR metagenomes (Fig. S1). We mapped SR over LR of the corresponding sample to assess the degree of sequence overlap between metagenomic samples (Fig. S2). From the total number of SR per sample, an average of 80% had a match in the corresponding LR time point. Similarly, LR with an SR match represented an average of 93% for the four time points. Thus, both sequencing technologies recovered equivalent microbial community fractions and sequence diversity, although LR-based metagenomics captured a higher GC content sequence space.

### Comparison of assemblies from SR and LR metagenomic samples

The total number of base pairs of assembled SR was, on average, more than four times the length of assembled LR (1.7 vs. 0.42 Gbp; see Table S2). The contigs recovered from LR metagenomes had a higher N50 of 86 kbp than the 1.2 kbp obtained from SR-derived contigs (500 bp cutoff; Fig. S3a and Table S2a). However, when these SR contigs were filtered using a 2.5 kbp length cutoff (which is the minimum length used for binning), the N50 increased to 7.5 kbp on average (Table S2b). More than ~84% of SR and ~66% of LR mapped back to their respective contigs (Table S2a). As expected for SR metagenomes, the sequencing depth of contigs reached higher values compared to LR for all dates (avg. ~2400x vs. 500x). However, average values were higher for LR than SR (~10x vs. 4.4x; Fig. S3b), indicating a more even distribution of sequencing depth values for the LR technologies. Thus, assembled LR reads provide longer contigs than those from SR metagenomes.

Using LR reads in SR hybrid assemblers can increase the length of recovered contigs [41, 42]. In the libraries analyzed here, N50 values increased, on average, from 1.2 to 2.3 kbp (500 bp cutoff) and 8.2 kbp (2.5 kbp cutoff; Table S2c). Nonetheless, the recovered contigs comprised, on average, ~17% fewer bases than those only using SR. Thus, adding LR libraries to the assembly increased the contig size but not the total length of the assembled space. Previous research has extensively documented and compared hybrid assembly approaches in metagenomic samples [10, 11, 41, 42]. Therefore, we focused on contrasting SR and LR platforms for MAG recovery.

### Gene predictions in unassembled and assembled long-read metagenomes

Among the most notable advantages of LR-based metagenomics is preserving long genomic regions on a single read. Nonetheless, indels can cause a frameshift in predicted protein sequences from LRs [43], especially when using unassembled sequences. Thus, we compared the length of predicted protein sequences from unassembled LR metagenomes to those in a comprehensive database. Commonly used tools, such as Prodigal [38], resulted in a higher fraction of smaller predicted protein sequences (length of predicted protein/length of reference protein; median = 0.93, IQR = 0.525, Fig. S4a). Gene prediction tools that can better manage errors in sequencing, such as FragGeneScan [37], resulted in a tighter distribution of predicted protein sequences (median = 1, IQR = 0.164, Fig. S4a). Thus, error-correction gene prediction tools are advantageous for indels correction in unassembled CCS reads. Prodigal was preferred when working with assembled reads (Fig. S4b, c). The distribution of predicted protein lengths compared to the lengths of references was more spread in SR (median = 0.983, IQR = 0.45) than LR metagenomic samples (median = 1, IQR = 0.052).

### Comparing SR and LR technologies for recovering MAGs from the same DNA sample

**Fig. 1:**
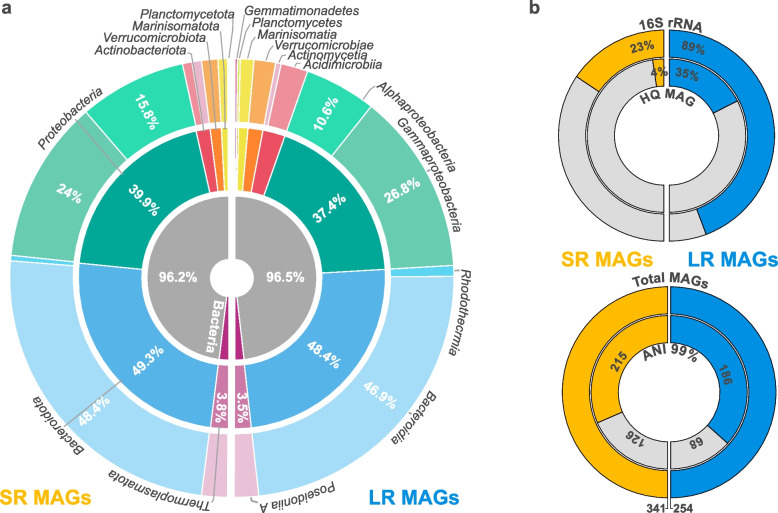
Metagenome-assembled genome recovered using short- and long-read metagenomic technologies. **a** Taxonomic profiles at the domain level (innermost ring), phylum level (internal ring), and class (external ring) levels for SR and LR MAGs recovered from all individual samples. **b** (Top) Detected 16S rRNA genes sequences (external ring) and high-quality MAGs (completion > 90%, contamination < 5%, 23S rRNA, 16S rRNA, 5S rRNA, > 18 tRNA, internal ring) recovered using SR and LR metagenomes. (Bottom) Total MAGs (external ring) and the number of de-replicated MAGs at the 99% ANI threshold from SR and LR metagenomes

The main goal of this study was to compare the capabilities of both SR and LR sequencing technologies for the recovery of bacterial and archaeal genomes (i.e., MAGs). First, we summarize the parallel comparisons of MAGs recovered from the same DNA extractions sequenced using SR and LR technologies. A total of 341 and 254 MAGs were reconstructed from the four SR and LR metagenomic samples, respectively (Fig. 1a). Remarkably, 88% of the MAGs recovered from LR metagenomic samples carried at least one copy of the 16S rRNA gene, contrasting with only 23% of the SR MAGs (Fig. 1b). Nonetheless, more 16S rRNA genes could be expected if a higher 2.5 kbp contig cutoff is used for the binning of contigs, although, at the expense of higher MAG fragmentation. No significant differences in completion were observed between all recovered LR and SR MAGs. However, all recovered LR MAGs were less contaminated than SR MAGs (Table S3; *p* < 0.05). Consistently, 35% (89/254) of the LR-derived MAGs meet the MIMAGs [44] high-quality criterion, whereas only 3.5% (12/341) of the SR MAGs follow under the same quality thresholds. The genome-based taxonomic composition was similar for both sequencing technologies (Fig. 1a). For instance, *Bacteroidia* (~ 47%) and *Gammaproteobacteria* (~ 25%) were the dominating groups in MAGs from both sequencing approaches, in agreement with the taxonomic profile of blooms from previous years [13].

**Fig. 2:**
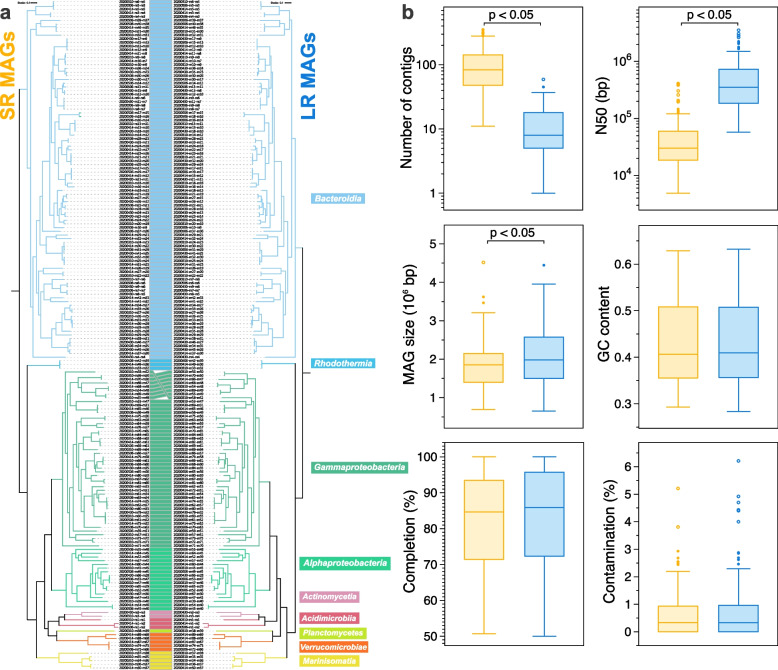
Comparisons between pairs of MAGs sharing ≥ 99% ANI. **a** Phylogenetic reconstructions of MAG pairs using short- and long-read metagenomic samples. MAGs sharing ≥ 99% ANI recovered from LR and SR metagenomes were used to generate a maximum-likelihood tree based on a group of 120 conserved genes. Colors represent different classes determined in GTDB-tk. **b** MAG pairs recovered from SR and LR metagenomes sharing ≥ 99% ANI compared according to genome size contigs, N50, number of contigs, GC content, completeness, and contamination

To compare genomic statistics of MAGs representing the same populations recovered from the same sample using both sequencing platforms, we generated pairs of genomes based on ANI ≥ 99% (Fig. 2). A parallel comparison of phylogenetic reconstructions derived from SR and LR MAGs was congruent, and a similar topology was observed (Fig. 2a). MAGs sharing ANI ≥ 99% were, for almost all cases, placed in similar positions in the tree. LR MAGs were composed of ~ 10 times fewer contigs than their ANI 99% SR counterpart (median = 8 vs. 83, *p* < 0.05 test; Fig. 2b). N50 values were also more than 11 times higher for LR MAGs (median ~ 350 kbp vs. ~ 30 kbp, *p* < 0.05). Interestingly, MAGs recovered from LR metagenomes were slightly longer than those from SR (median = 1.98 Mbps vs. 1.85 Mbps, *p* < 0.05). LR-derived MAGs also carried more predicted genes than the SR MAG pair (median = 1872 vs. 1704, *p* < 0.05; Fig. S5a). However, the differences in the number of predicted genes were not associated with particular taxonomic groups. This difference is likely a consequence of longer contigs built from reads containing complex regions not represented in SR, which are harder to reconstruct using SR approaches. Nonetheless, other statistics such as GC content, completion, and contamination were similar between the pair of MAGs.

### Differences in abundance and diversity in SR- and LR-derived MAGs

**Fig. 3:**
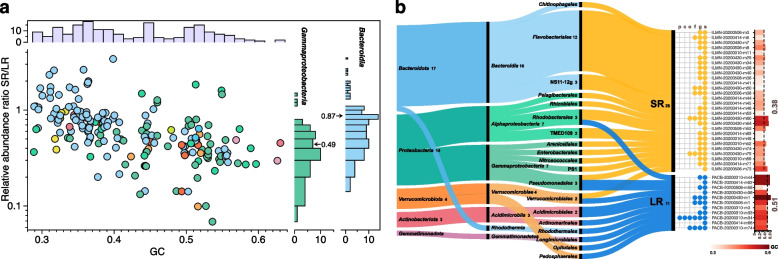
Differences between sequencing technologies at the MAG level. **a** The left plot compares the quotient between relative abundances of MAG pairs in SR and LR metagenomes determined at their respective sample of origin versus the GC content of the genome. The right histograms show the distribution of the quotient values for *Bacteroidia* and *Gammaproteobacteria* MAGs (the arrows indicate median values). The top histogram shows the distribution of GC content for all MAG pairs compared. Colored dots represent the assigned classes and follow the same palette as previous figures. **b** Unique species-level MAGs recovered from SR and LR. The horizontal flow diagram shows the taxonomic affiliation for unique species determined at the 95% ANI level in GTDB-tk. The dots summarize the novelty taxonomic level for each of the MAGs (p = phylum, c = class, o = order, f = family, g = genus, s = species). The bars on the right side represent the average GC content for the contigs of each MAG

Overall, the comparison of relative abundance determined for the paired MAGs was correlated between sequencing technologies (Fig. S6). Nonetheless, divergences from the expected abundances for the pair of MAGs were evident when inspected at each time point, especially for the three samples obtained during the bloom (April 14, 2020, April 30, 2020, and May 6, 2020). Unlike the March sample, the linear regression for the samples in April and May was less predictive, and a higher abundance for LR MAGs was evident (Fig. S6). These differences are primarily due to the increased representation (i.e., sequencing depth) of populations with higher GC content, such as *Gammaproteobacteria*, compared to *Bacteroidia* in LR metagenomes (Fig. 3a and Fig. S5b). However, a similar relative abundance for main taxonomic groups was observed between SR and LR MAGs when all, or those detected in both technologies, were determined (Fig. S5c, d). The exception was the higher abundances of *Bacteroidia* MAGs belonging in SR metagenomic samples at the last time point, in agreement with the observations at the MAG level. Thus, genomes with high GC content, such as *Gammaproteobacteria* and *Acidimicrobiia*, have increased sequencing depth in LR metagenomes. In contrast, *Bacteroidia* MAGs (with lower GC content) have a comparatively higher sequencing depth in SR metagenomic samples. Discrepancies in relative abundance between technologies due to GC content cannot be discarded for other groups likely not well captured in this analysis.

A critical aspect of ecological inferences based on recovered MAGs is determining whether both technologies can retrieve comparable units of diversity or species. To reduce the redundancy of MAGs belonging to the same populations captured from each time point, we de-replicated and selected representative MAGs at 99% ANI. Although 99% ANI is a high cutoff [45], de-replicated genomes resulted in 16% more MAGs from SR metagenomic samples (216 SR and 187 LR MAGs). This higher diversity is due to the higher sequencing effort (or the number of base pairs) obtained from SR compared to LR sequencing runs. When comparing the taxonomic affiliation of MAGs, 28 and 11 species were detected only in SR and LR MAGs, respectively. Interestingly, among these groups of uniquely recovered species, LR MAGs had an average higher GC content when compared to the SR MAGs (0.51 vs. 0.38; Fig. 3b). These differences likely reflect the inherent genomic characteristics of the groups enriched within each sequencing technology [46]. Biases in low and high GC content spaces are recognized for SR technologies [47], while reports on LR approaches have noted anywhere from minimal GC biases in mock communities [8] to a higher recovery of high GC content sequences in metagenomes [46, 48, 49]. According to our results, most SR-only species belonged to *Bacteroidia*, *Alphaproteobacteria*, and *Gammaproteobacteria*, whereas for LR-only species, *Acidiimicrobiia*, and *Verrucomicrobiae* were the two major classes. At the genus level, ~ 79% (22/28) and ~ 64% (7/11) of the unique MAGs belonged to genera only recovered in SR and LR metagenomes. At the phylum level, the exception was a *Gemmatimonadota* MAG with 63% GC content only recovered in LR metagenomes (PACB-20200310-m34, Fig. 3b). This *Gemmatimonadota* MAG had an abundance of 0.04% of the total community (truncated average sequencing depth, TAD80 = 3.77x, breadth of coverage = 88.34% at 1x TAD80) in the corresponding SR metagenome (March 10, 2020). These results suggest that SR approaches failed in recovering this MAG due to low sequencing depth and high GC content of the target genome.

To test the effect of low sequencing depth and breadth of the coverage in the recovery of unique species, we performed a cross-platform mapping of SR and LR to the MAGs, representing unique species recovered with the opposite technology (Fig. S7). For the most part, the cross-mapping of SR and LR on unique MAG species resulted in low sequencing depth (median = 6.4 vs. 2.8) and breadth of the coverage (median = 96 vs. 88.3%) for SR and LR technologies. Thus, uniquely detected species in each dataset are likely due to a combined effect of differences in GC content [46] and sequencing depth between technologies.

### Other considerations when choosing LR technologies

Currently, PacBio LR shotgun metagenomics is of higher cost per Gbp than SR (~ 2.4 times higher for our project, a further breakdown of costs is available in Table S1). The cost per Gbp of Nanopore is currently between Illumina and PacBio. Nanopore technologies offer the affordability and benefits of recovering longer reads or the possibility of including short technologies for the better recovery of high-quality MAGs [4, 10, 50]. Nonetheless, sequencing error and read lengths should be considered when selecting between LR technologies [4]. Despite the cost differences, the results presented here can guide researchers in deciding if LR metagenomics would be beneficial over SR approaches.

The current stage of algorithms and approaches for LR metagenomics is still limited compared to the large toolbox of SR technologies. While the methodology used here reflects the most appropriate tools and algorithms available at the time, we recommend that future studies pursue a critical assessment of newer approaches [12] when using LR techniques. The dataset presented here can also serve as a reference for testing and comparing algorithms and approaches for shotgun LR metagenomic sequencing.

## Conclusions

Our results highlight that switching from SR to LR metagenomic sequencing for microbial community analyses would still capture similar taxonomic composition from population genomes but recover higher-quality MAGs. Nonetheless, SR technologies offered more sequenced bases (e.g., three more times base pairs on average) than LR sequencing on single runs. This higher sequencing effort also translated into a higher number of dereplicated MAGs compared to LR metagenomic samples (i.e., a higher diversity of population genomes). This observation is relevant when the goal is to recover low-abundant organisms. Our work indicates a strongly decreased genome fragmentation and increased recovery of 16S rRNA genes in LR MAGs. These two features translate into better preservation of the order of genes in unassembled LR or LR-derived contigs. For instance, the generation of 16S rRNA probes for fluorescence in situ hybridization for single-cell identification and quantification. Even though a high fraction of overlapping reads was detected between technologies, differences in GC content likely resulted in slight differences in the recovery and abundance of some population genomes.

## Supplementary Information

**Additional file 1: Figure S1.** Average coverage and sequence diversity for SR and LR metagenomic samples. a. The estimated abundance-weighted average community coverage as determined in Nonpareil for each SR and LR metagenomic sample. For LR metagenomes, we first selected ~250 bp fragments from each LR and then used them to generate a model. Coverage was predicted from generated models and the original sequencing effort b. Sequence diversity (total diversity; *N*_*d*_) as defined in Nonpareil. **Figure S2.** Read overlapping between short- and long-read metagenomic samples. The bars show the mapping of SR on LR for each time point. The dark shades indicate the mapped fractions, and the light shades show unmapped SR and LR. **Figure S3.** Assembly statistics for contigs generated using short- and long-read metagenomic samples. a. N statistics for contigs generated using SR and LR metagenomic samples. b. Distribution of sequencing depth (x-axis) for contigs generated using SR and LR metagenomic samples. **Figure S4.** Distribution of predicted protein lengths using different gene prediction tools vs. best hit match in UniProt TrEMBL. a Only predicted proteins >= 100 amino acids were used for all comparisons. The boxplots show the quotients between the length of predicted proteins using FragGeneScan (FGS), MetaGenemark, and Prodigal and the best match in UniProt TrEMBL for the unassembled long-reads of the 2020-03-10 sample. b. Distribution of predicted protein lengths from contigs vs. best match in UniProt TrEMBL for the 2020.03.10 LR sample. **Figure S5.** Statistics for pairs of MAGs recovered from SR and LR metagenomes. a. Difference between the number of predicted genes in MAG pairs recovered in SR and LR metagenomic samples. The boxplot in the lower right corner summarizes the comparison of predicted genes for MAG pairs. b. GC content for pairs of SR and LR MAGs colored according to their class taxonomic affiliation. The right side of the plot shows histograms for the distribution of GC content values of MAGs belonging to *Bacteroidia* and *Gammaproteobacteria* class levels. The thick black line depicts the median value of the distribution. c,d. Relative abundance for all MAGs recovered (c) and pairs of 99% ANI MAGs (d). Colors represent the taxonomic affiliation of MAGs according to GTDB-tk. **Figure S6.** Relationship between relative abundances of MAG pairs in SR and LR metagenomes for each time point. **Figure S7.** Comparison of the breadth of the coverage vs. sequencing depth for cross-mapping of reads. The figures summarize the mapping of (a) short-reads on long-read derived MAGs and (b) long-reads on short-read MAGs. Uniquely detected species are colored according to the inferred taxonomy. The rest of the points represent MAG species detected in both technologies. The dotted purple line represents the expected breadth of coverage for a given level of sequencing depth, according to Lander and Waterman (1998). The letters indicate the novelty taxonomic level for each of the MAGs (p=phylum, c=class, o=order, f=family, g=genus, and the species level is omitted for clarity).

**Additional file 2: Table S1.** General sequence statistics for unassembled short- and long-read metagenomic samples. **Table S2.** General statistics for assembled reads. Summary for assemblies using 500 bp (a) and 2,500 bp (b) contig length cutoffs. Assembly statistics for the hybrid assembly approach (c). **Table S3.** List of generated MAGs. Names and general taxonomic classification for the MAGs used in this work. Accession numbers (ENA), completion, and contamination values for each MAG are also provided.

## Data Availability

All sequence data was deposited under the project PRJEB52999 in ENA and https://gitlab.mpi-bremen.de/lorellan/ilmn-vs-pacb-helgoland.
