# Supplementary material for: Comparing genomes recovered from time-series metagenomes using long- and short-read sequencing technologies
Source: Microbiome. 2023 May 13;11:105. doi: 10.1186/s40168-023-01557-3 (PMC10182627; doi:10.1186/s40168-023-01557-3)

Supplementary Figure 1

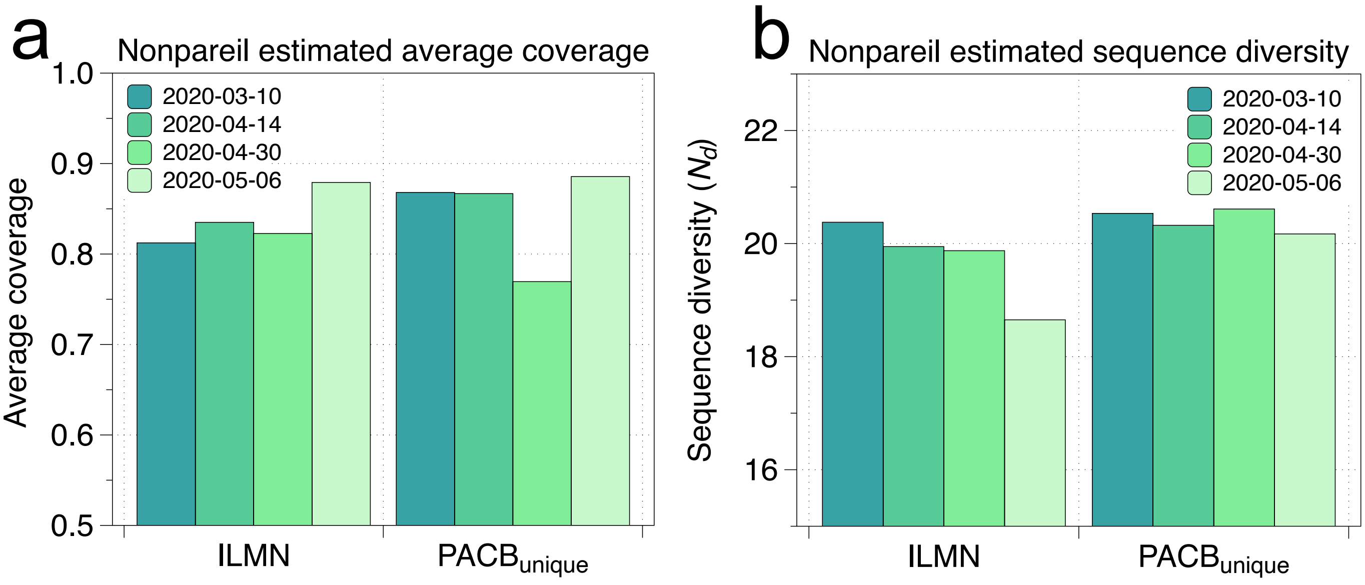

Supplementary Figure 2

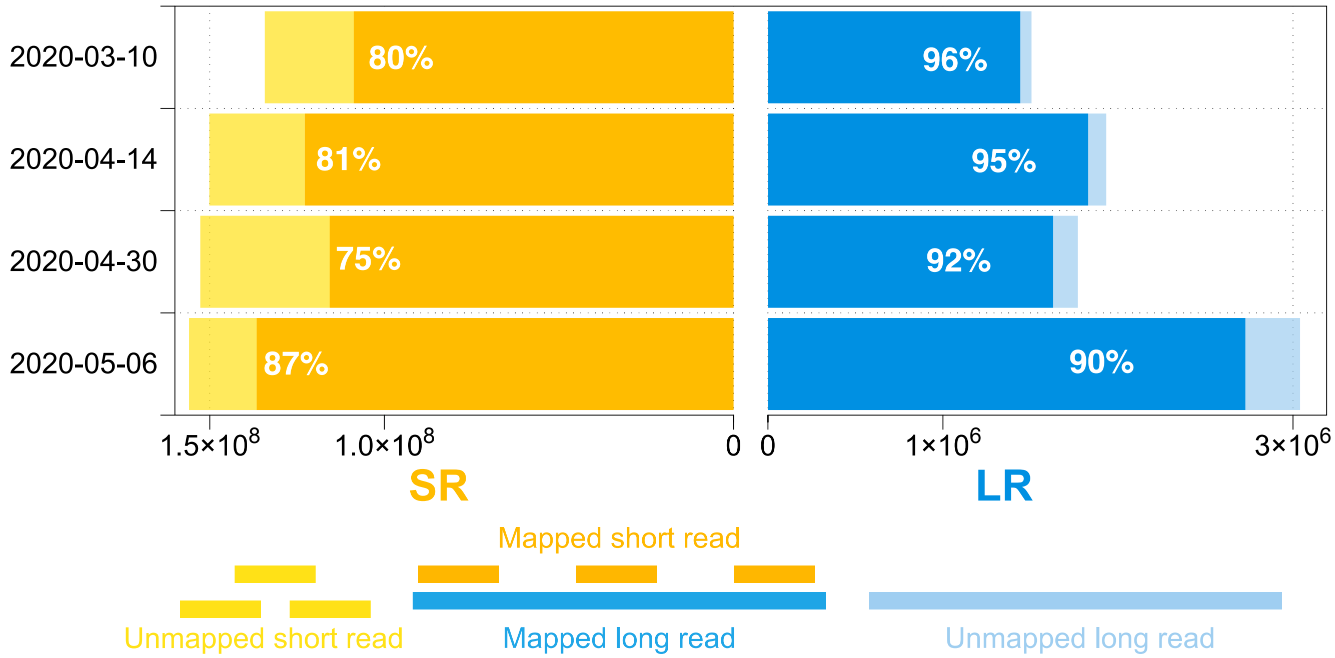

Supplementary Figure 3

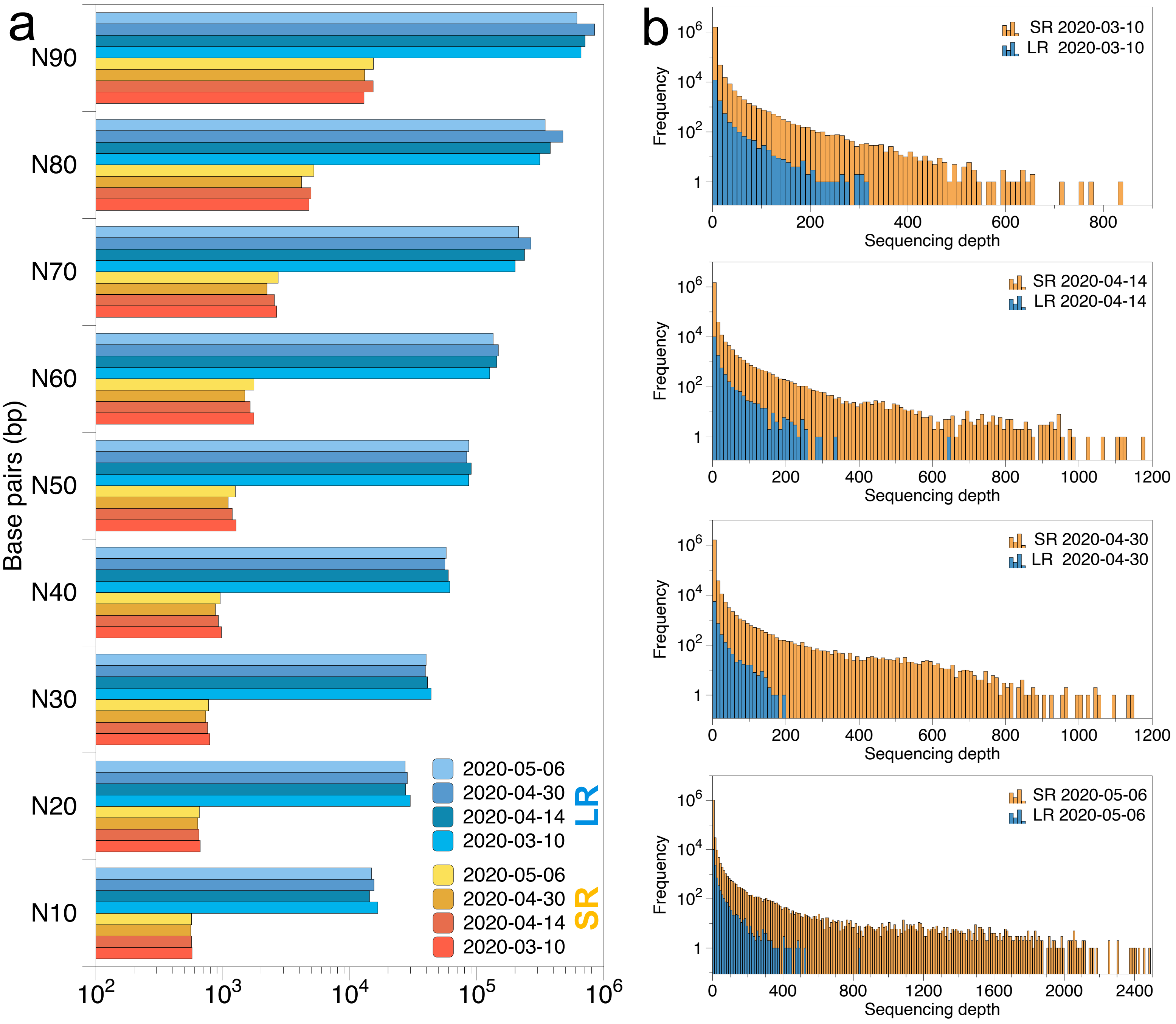

Supplementary Figure 4

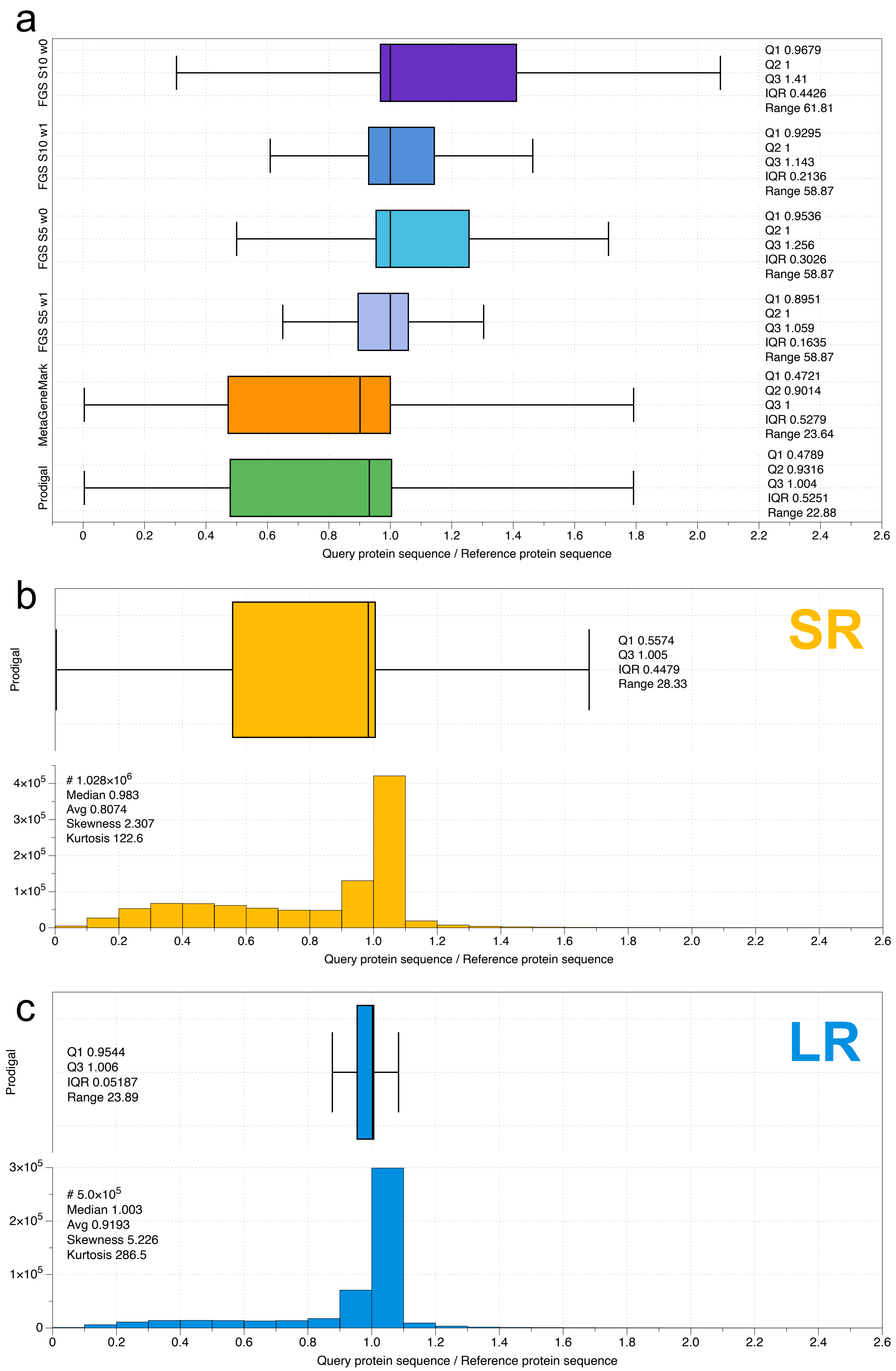

Supplementary Figure 5

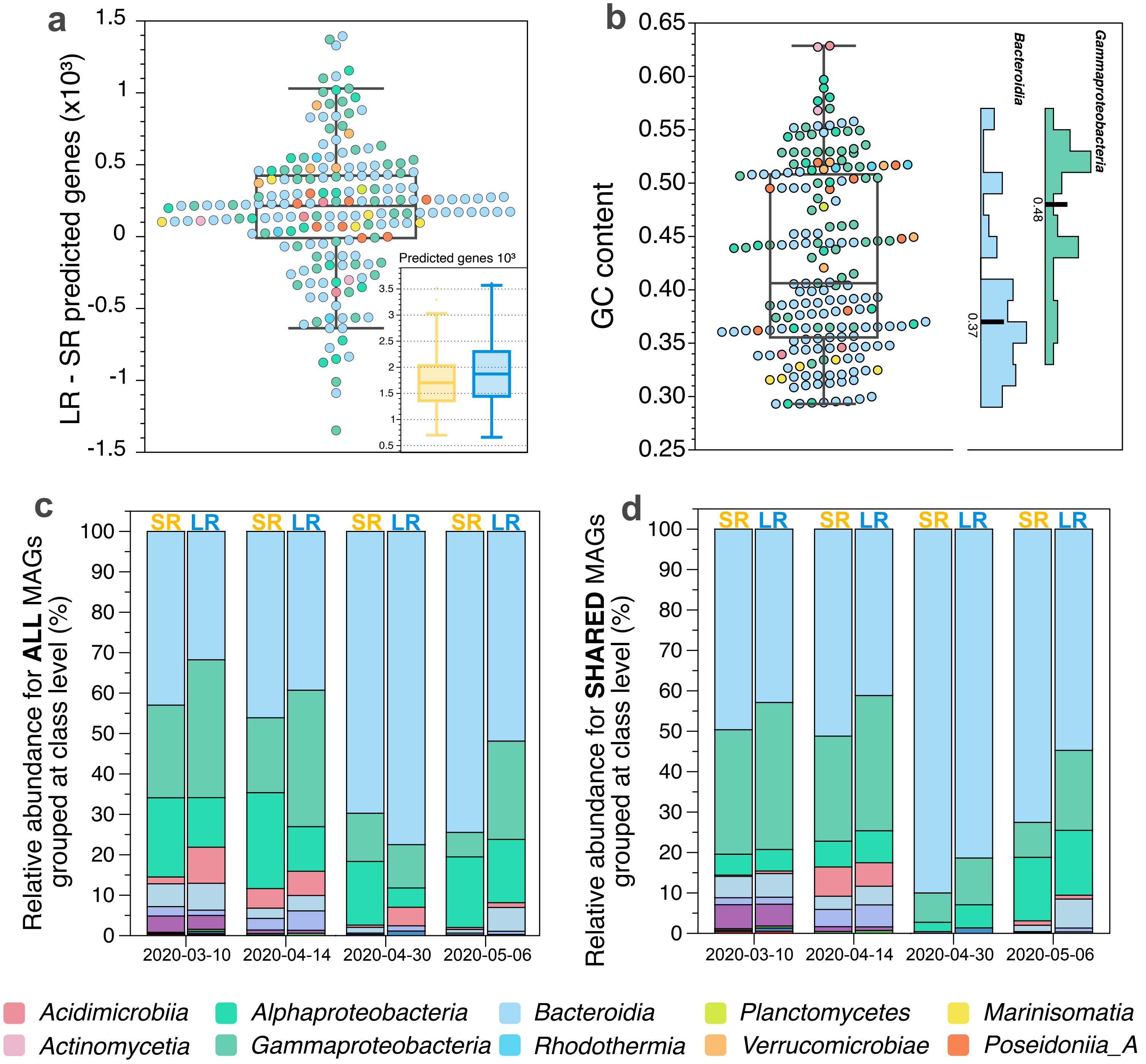

Supplementary Figure 6

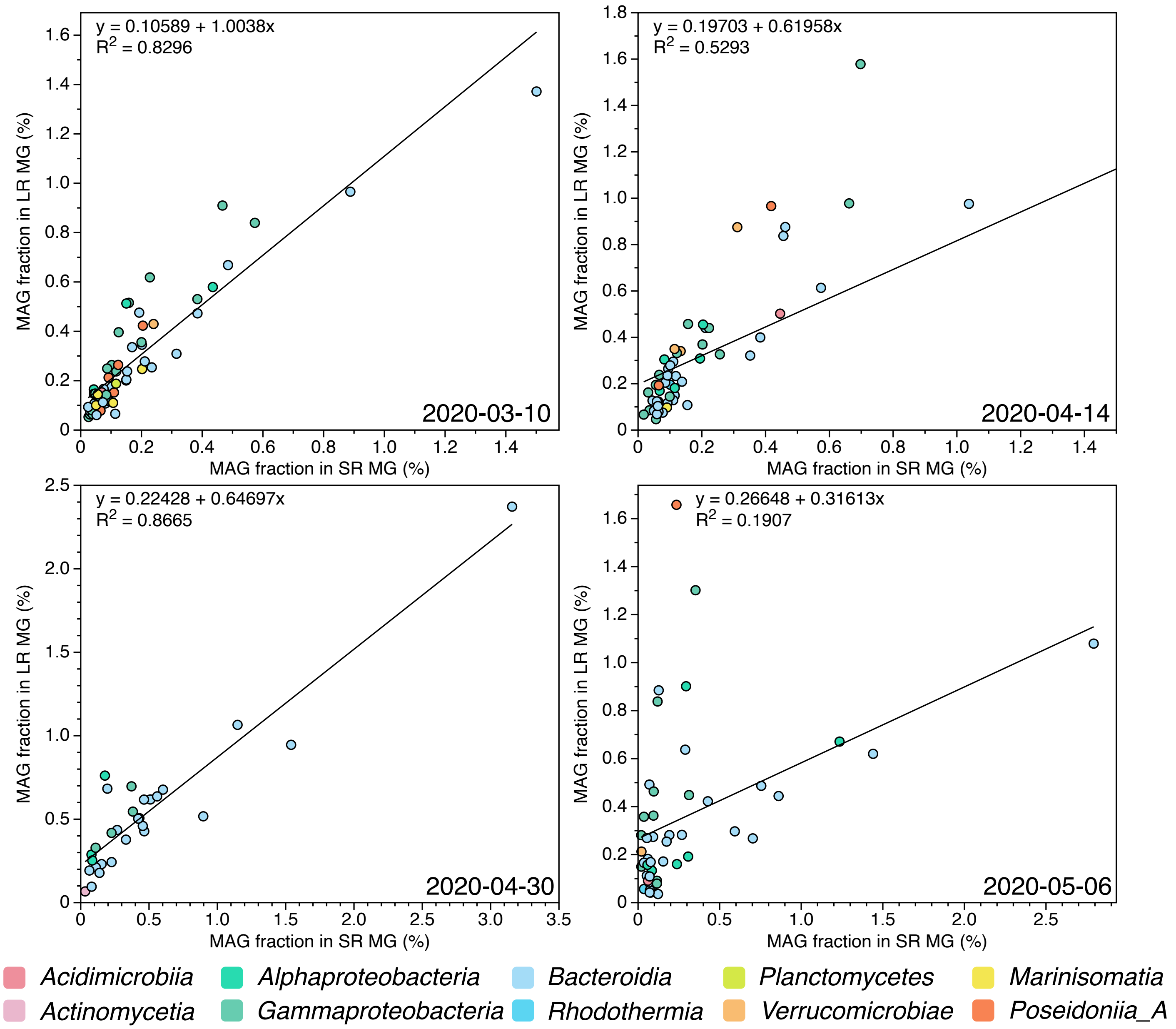

Supplementary Figure 7

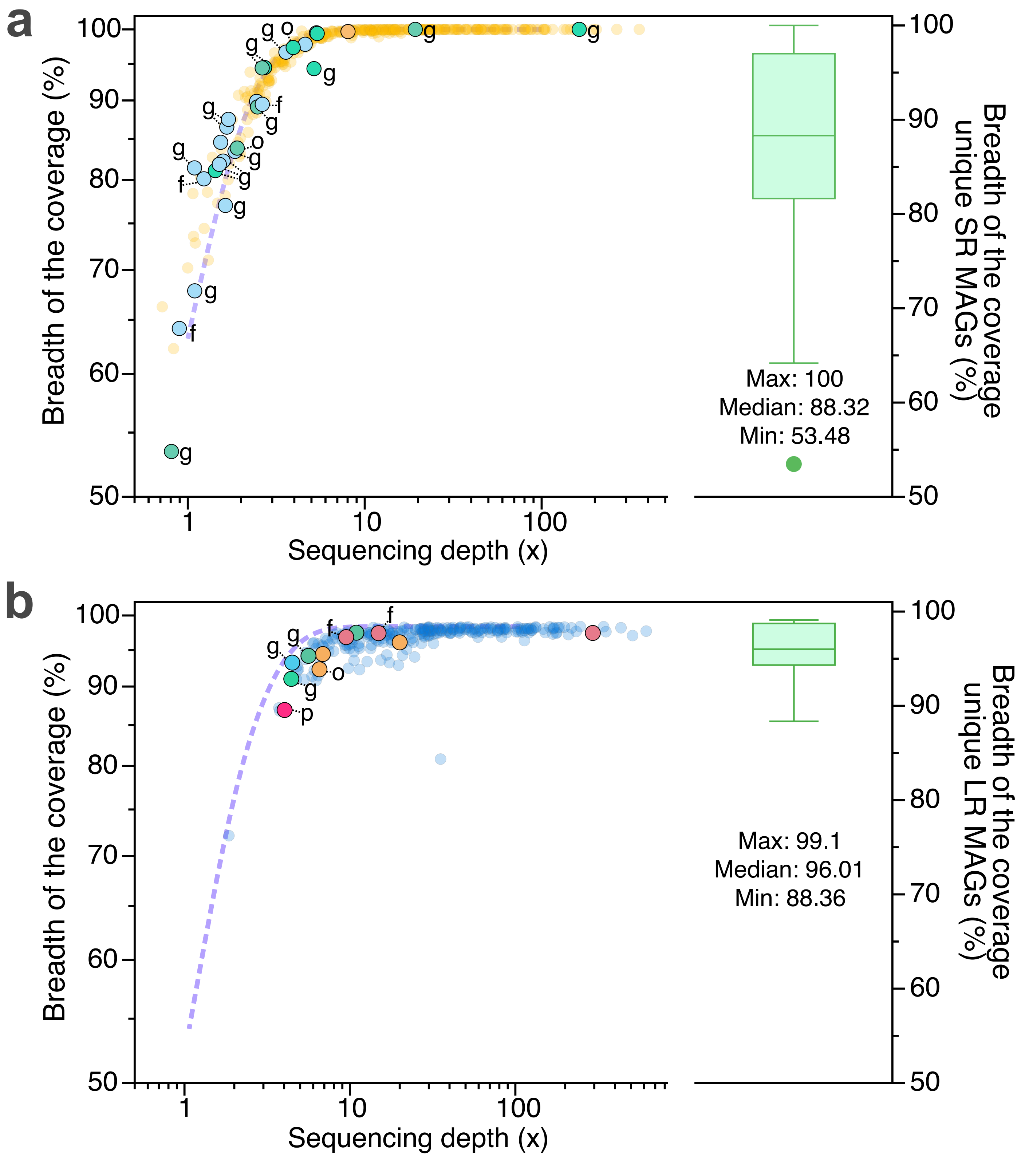

Supplement: Supplementary file 1 — Additional file 1: Figure S1. Average coverage and sequence diversity for SR and LR metagenomic samples. a. The estimated abundance-weighted average community coverage as determined in Nonpareil for each SR and LR metagenomic sample. For LR metagenomes, we first selected ~250 bp fragments from each LR and then used them to generate a model. Coverage was predicted from generated models and the original sequencing effort b. Sequence diversity (total diversity; Nd) as defined in Nonpareil. Figure S2. Read overlapping between short- and long-read metagenomic samples. The bars show the mapping of SR on LR for each time point. The dark shades indicate the mapped fractions, and the light shades show unmapped SR and LR. Figure S3. Assembly statistics for contigs generated using short- and long-read metagenomic samples. a. N statistics for contigs generated using SR and LR metagenomic samples. b. Distribution of sequencing depth (x-axis) for contigs generated using SR and LR metagenomic samples. Figure S4. Distribution of predicted protein lengths using different gene prediction tools vs. best hit match in UniProt TrEMBL. a Only predicted proteins >= 100 amino acids were used for all comparisons. The boxplots show the quotients between the length of predicted proteins using FragGeneScan (FGS), MetaGenemark, and Prodigal and the best match in UniProt TrEMBL for the unassembled long-reads of the 2020-03-10 sample. b. Distribution of predicted protein lengths from contigs vs. best match in UniProt TrEMBL for the 2020.03.10 LR sample. Figure S5. Statistics for pairs of MAGs recovered from SR and LR metagenomes. a. Difference between the number of predicted genes in MAG pairs recovered in SR and LR metagenomic samples. The boxplot in the lower right corner summarizes the comparison of predicted genes for MAG pairs. b. GC content for pairs of SR and LR MAGs colored according to their class taxonomic affiliation. The right side of the plot shows histograms for the distribution of G [file 40168_2023_1557_MOESM1_ESM.pdf]
