# Supplementary material for: Comparing genomes recovered from time-series metagenomes using long- and short-read sequencing technologies
Source: Microbiome. 2023 May 13;11:105. doi: 10.1186/s40168-023-01557-3 (PMC10182627; doi:10.1186/s40168-023-01557-3)
Supplement: Supplementary file 2 — Additional file 2: Table S1. General sequence statistics for unassembled short- and long-read metagenomic samples. Table S2. General statistics for assembled reads. Summary for assemblies using 500 bp (a) and 2,500 bp (b) contig length cutoffs. Assembly statistics for the hybrid assembly approach (c). Table S3. List of generated MAGs. Names and general taxonomic classification for the MAGs used in this work. Accession numbers (ENA), completion, and contamination values for each MAG are also provided. [file 40168_2023_1557_MOESM2_ESM.zip › Table_S2.pdf]

A

		Short-reads (Illumina contigs >= 500 bp)				Long-reads (PacBio contigs >= 500 bp)			
		2020.03.10	2020.04.14	2020.04.30	2020.05.06	2020.03.10	2020.04.14	2020.04.30	2020.05.06
Total contigs		1,682,426	1,627,889	1,734,416	1,114,338	17,438	15,061	7,618	15,850
Total bases		1,967,773,335	1,848,464,003	1,885,000,287	1,292,832,160	577,202,325	436,643,008	209,763,359	448,277,543
N50		1,272	1,186	1,102	1,254	86,322	90,327	83,292	86,503
Average contig length		1,170	1,135	1,087	1,160	33,100	28,992	27,535	28,282
Longest contig		469,761	529,644	655,714	751,657	3,066,831	2,637,556	3,527,687	2,827,647
Sequencing depth of mapped reads to contigs	mean	4.0	4.0	3.8	5.6	9.1	10.4	8.7	13.3
	median	2.0	1.8	1.8	2.0	4.5	4.8	4.4	5.8
	max	1,334.2	1770.6	1,570.7	5,158.6	319.9	642.8	194.6	830.4
Mapped reads to contigs >500bp %		84.27	84.13	82.13	87.00	82.21	78.09	52.55	78.11
Mapped reads to MAG contigs %		18.24	18.12	25.90	30.13	45.46	41.13	32.87	49.85
Mapped reads to unique species %		0.23	0.23	2.34	0.44	1.22	0.32	0.29	0.36

B

	Short-reads (Illumina contigs >= 2,500 bp)				Long-reads (PacBio contigs >= 2,500 bp)			
	2020.03.10	2020.04.14	2020.04.30	2020.05.06	2020.03.10	2020.04.14	2020.04.30	2020.05.06
Total contigs	101,465	86,513	81,903	64,273	14,542	11,825	5,482	11,944
Total bases	615,389,865	561,754,207	523,681,389	410,376,770	573,661,476	432,424,685	207,051,823	443,167,233
N50	6,852	7,838	7,617	7,569	87,700	92,346	85,135	89,896
Average contig length	6,065	6,493	6,394	6,385	39,449	36,569	37,769	37,104
Longest contig	469,761	529,644	655,714	751,657	3,066,831	2,637,556	3,527,687	2,827,647

C

	Hybrid (contigs>=500 bp)			
	2020.03.10	2020.04.14	2020.04.30	2020.05.06
Total contigs	1,137,336	1,110,724	1,194,595	715,850
Total bases	1,706,846,858	1,533,246,769	1,458,739,904	1,120,253,276
N50	2,485	1,930	1,376	2,806
Average contig length	1,501	1,380	1,221	1,565
Longest contig	651,542	599,012	639,037	617,625

D

	Hybrid (contigs>=2500 bp)			
	2020.03.10	2020.04.14	2020.04.30	2020.05.06
Total contigs	112,959	90,667	70,689	78,901
Total bases	851,643,835	694,318,110	548,296,656	584,835,033
N50	9,492	10,045	10,683	9,353
Average contig length	7,539	7,658	7,756	7,412
Longest contig	651,542	599,012	639,037	617,625
